# Screening and preliminary validation of miRNAs with the regulation of hTERT in colorectal cancer

**DOI:** 10.3892/or.2015.3892

**Published:** 2015-04-01

**Authors:** YU-ZHOU QIN, XUE-CHENG XIE, HAI-ZHOU LIU, HAO LAI, HAI QIU, LIAN-YING GE

**Affiliations:** 1 Departments of Gastrointestinal Surgery, Tumor Hospital of Guangxi Medical University, Nanning, Guangxi 530021, P.R. China; 2 Departments of Experimental Research, Tumor Hospital of Guangxi Medical University, Nanning, Guangxi 530021, P.R. China; 3 Endoscopy Center, Tumor Hospital of Guangxi Medical University, Nanning, Guangxi 530021, P.R. China

**Keywords:** miRNAs, hTERT, colorectal cancer

## Abstract

The overexpression of human telomerase reverse transcriptase (hTERT) has been associated with the invasion and metastasis of colorectal cancer (CRC) and has received extensive attention, although the underlying mechanism involved remains unclear. The aim of the present study was to screen and preliminarily validate new tumor-suppressor microRNAs (miRNAs) that potentially inhibit hTERT expression and to assess its clinical significance. Screening for downregulated miRNAs in CRC tissues was performed by retrieving and analysing microRNA microarray data. miRNA candidates were then filtered by bioinformatics analysis. The expression of miRNAs candidates was verified by quantitative polymerase chain reaction in the CRC and corresponding normal tissues. Immunohistochemistry (IHC) was used for the detection of hTERT protein expression. Spearman’s correlation coefficient between miRNA candidates and hTERT protein expression was calculated (r) to identify hTERT-targeting miRNAs. A survival analysis was performed to assess the prognostic significance of hTERT-targeting miRNAs in CRC. Eight miRNAs with the potential to interact with hTERT were predicted: miR-29c-3p, miR-124-3p, miR-133a-3p, miR-133b, miR-138-5p, miR-150-5p, miR-378a-3p and miR-422a, respectively. Following detection of the miRNAs using RT-qPCR, miR-29c-3p was excluded. miR-138-5p and miR-422a were observed to potentially interact with hTERT (r=−0.362, P=0.001; r=−0.306, P=0.005, respectively). The Kaplan-Meier survival curves demonstrating high- vs. low-expression group of miR-422a showed a highly significant difference in CRC patients (P=0.024), which suggests that the downregulation of miR-422a was associated with a poorer prognosis. The results indicated that miR-138-5p and miR-422a potentially inhibited hTERT expression in CRC, and suggest a potential application of miR-422a in prognosis prediction and CRC treatment.

## Introduction

Eukaryotic chromosomes are composed of tandemly repetitive telomere sequences that protect the ends from damage and rearrangements. Telomere repeats are synthesized by telomerase, a ribonucleic acid (RNA)-protein complex (1). Telomerase enables cancer cells to achieve replicative immortality, which is one of the hallmarks of cancer (2). Telomerase activity is currently the most general molecular marker for the identification of human cancer and can be detected in 85–90% of all tumors (3). Human telomerase reverse transcriptase (hTERT), which confers the catalytic activity of telomerase, is the restricting factor for telomerase activity (4). Investigations have shown that hTERT has a significant role in cancer tumorigenesis, growth, migration and invasion (5). Clinical studies have also proven that the overexpression of hTERT is associated with cancer progression and poor outcomes, although the underlying mechanism remains unclear (6). The majority of studies have focused on the co-regulation of hTERT via transcriptional regulation, the presence or absence of various activators and repressors, as well as the epigenetic pathways of DNA methylation and histone modifications (7). Few studies aimed to examine the regulation of hTERT via post-transcription.

microRNAs (miRNAs) are thought to control gene expression at the post-transcriptional level by degrading or repressing target messenger RNAs (mRNAs) (8). miRNAs are a class of newly identified eukaryotes, highly conserved, with a length of ~18–24 nt endogenous non-coding single-stranded RNA. They have crucial regulatory functions in cell differentiation, proliferation, and apoptosis (9). Each miRNA has multiple target genes, with well over one third of human genes appearing to be conserved miRNA targets (10). Recent findings have shown that the altered expression pattern of miRNAs is involved in many forms of cancer as oncogenes and tumor suppressors, playing an important role in cancer occurrence, development and progression, especially in colorectal cancer (CRC) (11).

Three different miRNAs (miR138 in human anaplastic thyroid carcinoma cell lines, miR-1207 and miR1266 in gastric cancer) have been identified that may influence hTERT expression (12,13). Since a miRNA can affect hundreds of target gene regulation, a gene can also be affected by molecular regulation of multiple miRNAs. Therefore, other miRNAs may be involved in the post-transcriptional regulation of hTERT.

Through retrieval of microRNA microarray data and biological information technology analysis, the expression of miRNAs with the potential to directly regulate hTERT was downregulated. Reverse-transcriptase quantitative polymerase chain reaction (RT-qPCR) was used to verify the expression of these miRNAs in the CRC and corresponding normal tissues. Subsequently, immunohistochemistry (IHC) was used in the detection of hTERT protein expression in the same samples. A preliminary validation of the miRNAs with the potential to regulate hTERT was obtained by identifying whether there is a negative relationship between expression of miRNAs and the hTERT IHC data.

## Materials and methods

### Patients, tissue samples and follow-up

The CRC and corresponding normal tissues were retrospectively recruited from 84 patients with CRC at the Tumor Hospital of Guangxi Medical University between 2007 and 2010. The present study was approved by the Institutional Review Board of the Guangxi Medical University. None of the CRC patients had undergone neo-adjuvant radiotherapy, chemotherapy or other treatment prior to surgery. Pathological diagnosis was also standardised and reviewed by committee criteria, chaired by a senior academic pathologist. Patient clinicopathological information and follow-up data of CRC were available from the documents of the hospital. The clinicopathological characteristics of patients are shown in Table I.

### Retrieval of microRNA microarray data

Gene Expression Omnibus (GEO) is a public functional genomics data repository supporting MIAME compliant data submissions. The GEO datasets were searched for relevant studies using the terms: (colon or rectum or rectal or colorectal) and (cancer or tumor or neoplasm). The study type was limited to ‘non-coding RNA profiling by array’, the organism was ‘homo sapiens’ and the attribute name was ‘tissue’. The dataset was included in our analysis when the certain criteria were met, i.e., the dataset was required to i) be microarray data expression of miRNAs in CRC; ii) be controlled studies in the CRC and corresponding normal tissue; iii) include ≥3 samples and iv) be available for download.

### Biological information technology

The common analysis methods of extract differentially regulated miRNAs from the dataset included previously were: significance analysis of microarrays (SAM), CyberT and rank products (RP), of which CyberT is the most commonly used analytical tool of computational biology, and bioinformatics analysis as its algorithm can be completely implemented in the linear models for the microarray data (Limma) package (14). In this study, the data were analyzed by using language R (3.2 versions). To test for differential expression, CyberT algorithm and the bayesian adjusted t-statistics from the Limma package statistical methods were used (15,16). A multiple-testing correction based on the false discovery rate (FDR) was performed.

The miRWalk (http://www.umm.uni-heidelberg.de/apps/zmf/mirwalk/) is a publically available comprehensive resource, hosting the predicted as well as the experimentally validated miRNA-target interaction pairs (17). miRWalk online software was used to predict miRNAs with the potential to interact with hTERT. The candidate hTERT-targeting miRNAs were selected for analysis in the subsequent experiments.

### Reverse transcriptase quantitative polymerase chain reaction

Reverse transcriptase quantitative polymerase chain reaction (RT-qPCR) was performed to detect the included miRNAs expression. Total RNA was extracted from tissue samples using a Qiagen miRNeasy Mini kit (Qiagen GmbH, Hilden, Germany) according to the manufacturer’s instructions. The total RNA was then eluted in a 30-*μ*l volume of elution buffer. RNAs were quantified by Nanodrop 2000 (PeqLab Biotechnology GmbH, Erlangen, Germany). RNA samples were converted to cDNA using miScript II RT kit (Qiagen). qPCR was performed in a total volume of 20 *μ*l reaction mixture containing cDNA product, specific primers for each miRNA (Invitrogen, Carlsbad, CA, USA), miScript Universal primer (Qiagen), SYBR-Green qPCR Master Mix (Thermo Fisher Scientific, Waltham, MA, USA) and nuclease-free water. The U6 snRNA gene was used as an internal control. The sequences of the specific forward primers are shown in Table II. PCR reactions were conducted at 95°C for 7 min, followed by 40 cycles of 95°C for 10 sec, and 60°C for 30 sec in a Mx3000P Real-Time Quantitative PCR system (Agilent Technologies, Inc., Santa Clara, USA). To minimize data variation in separate runs, paired cancer and corresponding normal tissues from the same patient were detected on the same runs. The Ct value was calculated using MxPro-Mx3000P software (Agilent Technologies) using the automatic threshold setting. The reactions were run in triplicate. Results were presented as the levels of expression following normalization to U6 using the 2^−Δ∆Ct^ method (18).

### Immunohistochemistry

Sections (4-*μ*m) were cut from the selected paraffin blocks and dried overnight at 37°C. The sections were dewaxed and rehydrated, exposed to 3% H_2_O_2_ solution for 10 min to block endogenous peroxidase and subjected to antigen retrieval in Tris-EDTA (pH 9.0). Subsequently, the slides were incubated with the rabbit monoclonal antibody against hTERT (1:100; Abcam, Cambridge, MA, USA), overnight at 4°C. Following three washes in 0.01 mol/l phosphate-buffered saline (PBS, pH=7.4), labeling was detected by adding a secondary antibody for 15 min at room temperature and diaminobenzidine (both from Maxim-Bio, Fuzhou, China) after washing in PBS again. The sections were then counterstained with hematoxylin, dehydrated, and mounted. Negative controls were conducted by replacing the primary antibody with PBS.

The sections were blindly and independently assessed microscopically by two well-trained pathologists. For the assessment of hTERT, five high-power fields in each specimen were randomly selected, and yellow or brown staining of the cytoplasm or nuclear was considered positive staining. The hTERT immunostaining score was calculated with a semi-quantitative scoring system as the intensity (0, no staining; 1, weak staining; 2, moderate staining; 3, strong staining) and the percentage (extent staining) of tumor cells that were stained (0, <10% of tumor cells stained; 1, 10–50% of positive cells; 2, >50 and <75% of positive cells; 3, >75% of positive cells). An overall score was obtained as the product of the intensity and distribution of positive staining (19). Cases with 0 points were considered to be negative (0), cases with a final score of 1–3 as weakly positive (1+), cases with a final score of 4–7 as moderately positive (2+) and cases with a final score of >7 as strongly positive (3+).

### Statistical analysis

Statistical analysis was performed using Statistical Program for Social Sciences (SPSS) software, version 16.0 (SPSS Inc., Chicago, IL, USA). RT-qPCR data were expressed as medians (IQR, interquartile range). Possible differences between the CRC and corresponding normal tissue groups were analyzed using the Wilcoxon’s signed-rank test. Correlations between the expression of hTERT proteins (immunohistochemical scores) with the expression of miRNAs were evaluated using the Spearman’s rank-order correlation coefficient. Survival curves were obtained by the Kaplan-Meier method. A comparison between curves was made using the log-rank test. The tests were performed as two-tailed and the level of significance was set as P<0.05.

## Results

### Identification of downregulated miRNAs through retrieval of microRNA microarray data and language R analysis

A total of eight datasets fulfilled the inclusion criteria for our analysis: GSE10259, GSE33127, GSE35602, GSE38389, GSE39845, GSE49246, GSE18392 and GSE35982, respectively. Subsequently, we used language R to analyze the eight included data. First, 173 significantly downregulated miRNAs were selected with log_2_FC<−0.5 and a P-value of <0.05 by analyzing language R. Second, according to the number of chip data source arrangement (highest score, 8 points and lowest score 1 point), 45 significantly downregulated miRNAs with a frequency of ≥2 were identified. An intersection of the 45 miRNAs and downregulated miRNAs provided from the published literature corresponding to the eight microarray data was performed. Thirty-two significantly downregulated miRNAs in CRC tissue were found.

### Identification of candidate hTERT-targeting miRNAs through biological information technology

Initially, 359 miRNAs were found by using miRWalk online software. Points ≥2 were selected, duplicate values were removed, and ultimately 125 hTERT regulation-related miRNAs were obtained according to the integral arrangement (results of the 1 point represent only one software prediction, with a maximum of 6 points and a minimum of 1 point). miRNAs were obtained by the intersection of the 32 significantly downregulated miRNAs and the 125 miRNAs associated with the regulation of hTERT. Eight miRNAs with the potential to interact with hTERT were predicted: miR-29c-3p, miR-124-3p, miR-133a-3p, miR-133b, miR-138-5p, miR-150-5p, miR-378a-3p and miR-422a, respectively.

### Validation of the 8 selected miRNAs by RT-qPCR

**Figure 1:**
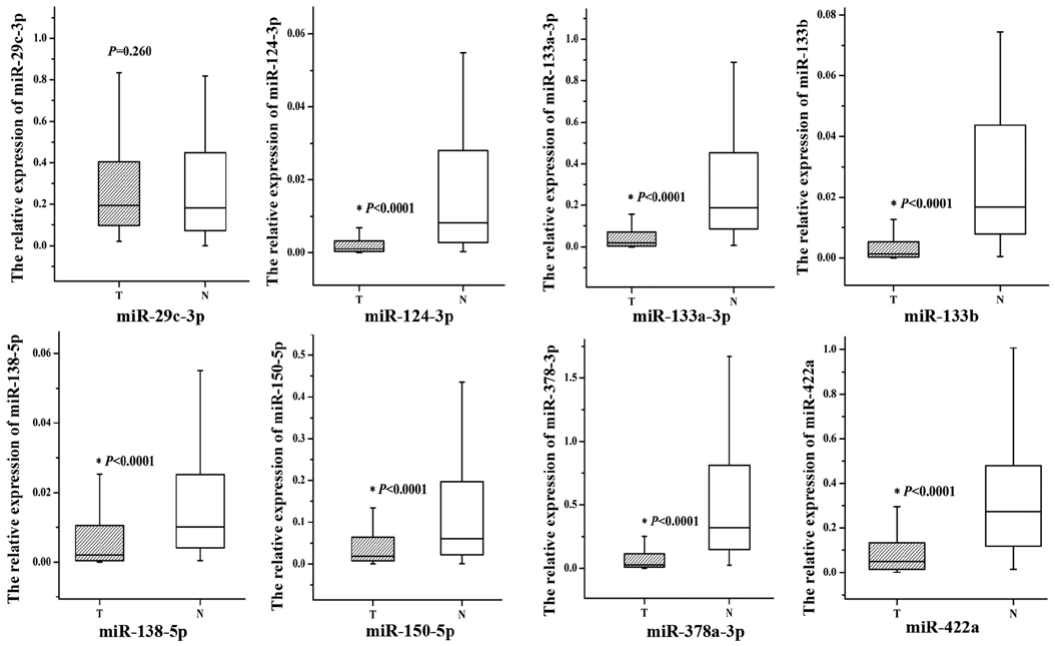
The relative expression levels 8 miRNAs in colorectal cancer and corresponding normal tissue group, respectively. ^*^Statistically significant.

**Figure 2:**
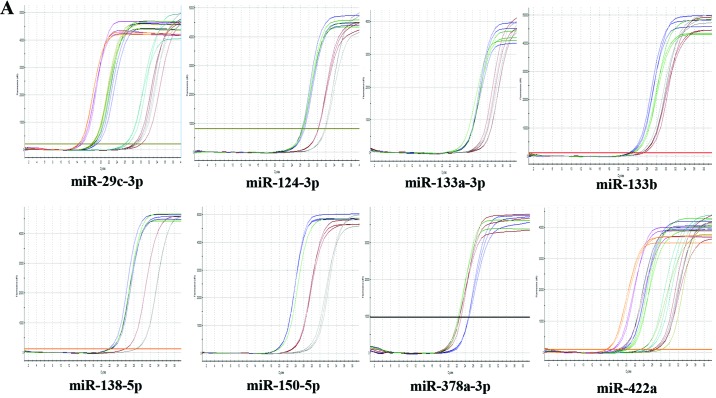

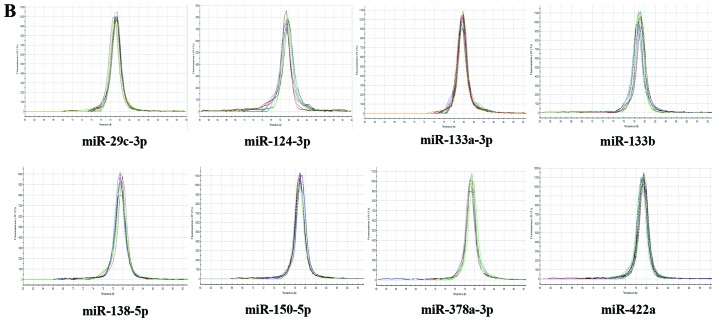

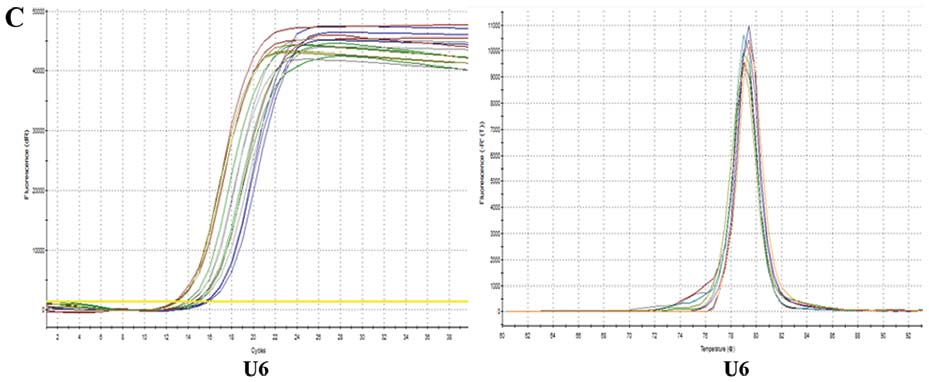
miRNAs and U6 snRNA expression in colorectal cancer and normal tissues by RT-qPCR. (A) The amplification curves of 8 miRNAs, respectively. (B) The corresponding melting curves analysis of 8 miRNAs demonstrates single peak. (C) The amplification curve and melting curve of U6 snRNA (internal control).

To validate the relative expression levels of 8 selected miRNAs in CRC and corresponding normal tissues, we performed qPCR experiments. The results revealed that the expression level in CRC tissues of miR-124-3p, miR-133a-3p, miR-133b, miR-138-5p, miR-150-5p, miR-378a-3p and miR-422a (P<0.0001 for all) were statistically significantly downregulated when compared with the corresponding normal tissues. However, there was no significant difference in the levels of miR-29c-3p (P=0.260, Fig. 1 and Table III). The amplification curves and the melting curves of 8 miRNAs and U6 in the qPCR phase are shown in Fig. 2.

### Immunohistochemical expression of hTERT protein in paraffin sections and the correlations with expression levels of the 7 verified miRNAs

**Figure 3:**
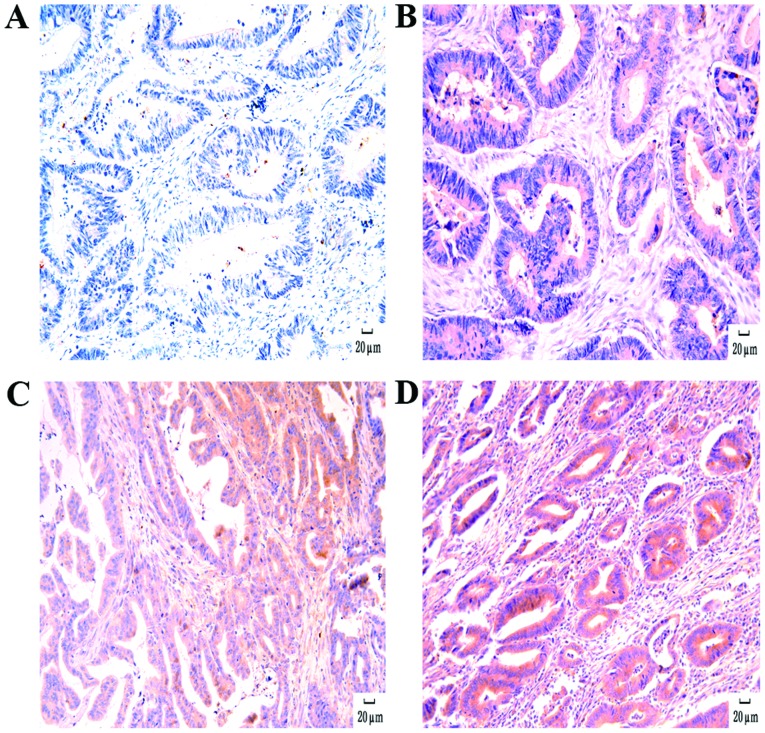
Representation of photomicrographs for hTERT expression in colorectal cancer tissues, respectively. (A) Negative expression (−). (B) Weakly positive (1+). (C) Moderately positive (2+). (D) Strongly positive (3+) (magnification, x200).

Positive hTERT inmunoreactivity was observed in cancer tissue of 60 patients, while in 24 patients it was negative. As shown in Fig. 3, the positive expression of hTERT was localized to the cytoplasm or nuclei in colorectal tumor cells. Spearman’s rank-order correlation coefficient between miRNAs and hTERT protein expression was calculated (r). As the frequency distribution for miRNAs in the group was non-parametric, the median (non-parametric distribution) was used as the cut-off level of expression to divide patients into the high- and low-expression groups for statistical testing. Our results suggested that hTERT protein showed a significant negative correlation with the expression levels of miR-138-5p (r=−0.362, P=0.001) and miR422a (−0.306, P=0.005), while the correlation between hTERT and other miRNAs (miR-124-3p, miR-133a-3p, miR-133b, miR-150-5p and miR-378a-3p) revealed no significant negative correlation (Table IV). Therefore, the downregulated expression of miR-138-5p and miR-422a potentially inhibited hTERT expression.

### Relationship between miR-138-5p and miR-422a expression and clinicopathological factors in CRC patients

For statistical testing, we primarily divided patients into the high- and low-expression groups according to the median value of each miRNA. The expression levels of miR-138-5p and miR-422a were compared between different cohorts depending on various clinicopathological characteristics (Table I). There were no significant variations in miR-138-5p between the subgroups regarding age, gender, tumor size, tumor site, differentiation, the depth of invasion, lymph-node metastasis and TNM stage. However, a statistically significant difference in miR-138-5p expression was observed with regard to distant metastasis (P<0.000). A significant difference in miR-422a expression was identified between the subgroups according to lymph-node metastasis (P=0.023). However, the data showed no significant association between miR-422a relative expression levels and other parameters.

### Downregulated miR-422a associated with shorter overall survival (OS)

**Figure 4:**
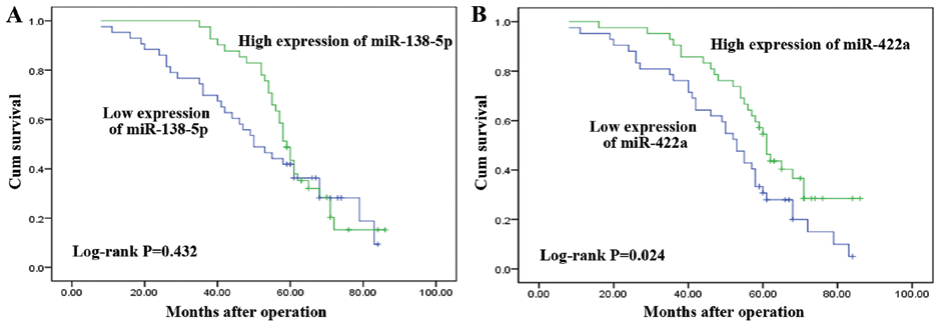
Overall survival curves of 84 patients with colorectal cancer according to whether they show high or low (A) miR-138-5p and (B) miR-422a levels.

The survival analysis of the 84 studied patients was implemented using information available from the clinical follow-ups. The median follow-up was 58 months (range, 8–86 months). At the end of follow-up, 62 patients succumbed, 22 patients remained alive, and the follow-up rate was 100%. Results of the Kaplan-Meier method and log-rank test showed that the OS of CRC patients with a high-expression miR-138-5p was longer than that of patients with low-expression miR-138-5p (the estimated median OS time was 59 and 50 months, respectively), albeit the difference was not statistically significant (P=0.432, Fig. 4A). However, patients with low-expression miR-422a had significantly poorer OS (P=0.024, Fig. 4B). The estimated median OS time was 61 months in the miR-422a high-expression group but 53 months in the miR-422a low-expression group. Our result suggested that a lower expression of miR-422a was associated with reduced OS in CRC patients.

## Discussion

CRC is the third most common cancer in humans and the mortality of CRC accounts for ~9% of all cancer deaths (20). Therefore, gaining a better understanding of the biological behavior of the tumor is critical to improve treatment strategies and patient outcomes. The dysregulation of miRNAs and hTERT have been suggested to play a crucial role in the regulation of tumorigenesis and metastases of various types of cancer. The finding that miRNAs influence cell proliferation, apoptosis, metabolism, and transformation in many subtypes of cancer at a post-transcriptional level, has led to great attention being focused on novel tumor-suppressor oncogene-targeting miRNAs.

In this study, we initially identified 8 miRNAs that were potentially involved in the regulation of hTERT by using a combination of retrieving and analysing microRNA microarray data and bioinformatics analysis. The expression levels of the 7 miRNAs (miR-124-3p, miR-133a-3p, miR-133b, miR-138-5p, miR-150-5p and miR-378a-3p, miR-422a) were found to be significantly decreased in 84 pairs of the CRC tissues when compared with their matched corresponding normal tissues using RT-qPCR. Our results suggest that hTERT protein showed a significant negative correlation with the expression levels of miR-138-5p (r=−0.362, P=0.001) and miR422a (−0.306, P=0.005). Thus, miR-138-5p and miR-422a may be more likely to inhibit hTERT expression potentially compared to the remaining 5 miRNAs.

To the best of our knowledge, this is the first study to screen and validate preliminarily miRNAs with regulation of hTERT in CRC. Other studies have identified 3 different miRNAs that influence hTERT expression. miR-138 was the first demonstrated hTERT-targeting miRNA. Mitomo *et al* (13) have shown that the overexpression of miR-138 induced a reduction in hTERT protein expression in human anaplastic thyroid carcinoma. Additionally, using luciferase reporter assay those authors confirmed target specificity between miR-138 and the hTERT 3′-untranslated region. Chen *et al* (12) determined that miR-1207-5p and miR-1266 interact with the 3′UTR of hTERT and suppress gastric cancer growth and invasion by targeting hTERT. However, in addition to the main mechanism described above, miRNAs can also regulate the expression of hTERT through influence of other transcription factors. Wang *et al* (21) found that miR-21 regulates hTERT expression mediated by STAT3, thereby controlling glioblastoma cell growth. Moreover, the scope of functional miRNA-mRNA interactions have been expanded from RNA 3′UTRs to include the coding regions of the targeted RNAs (22). These mechanisms described above offered possible interpretations of the observed negative statistical association between the downregulated miR-138-5p and miR-422 and the overexpression of hTERT protein.

In contrast to hTERT promoting tumor metastasis, miR-138 and miR-422a have been found to be potential tumor suppressors in certain types of cancer. It has been shown that miR-138 may have an effect on tumor metastasis by targeting SOX4 and HIF1a in ovarian cancer and targeting MMP2/MMP9 in cholangiocarcinoma (23,24). Downregulation of miR-138 promotes metastasis by directly targeting TWIST2 and is associated with lymph-node metastasis, distant metastasis, and predicted poor prognosis in CRC (25). Previous findings suggested that miR-422a may play a protective role against CRC, which was shown by its decreased expression in CRC when compared to normal tissue (26). Furthermore, miR-422a can suppress tumor cell proliferation by inhibiting related pathways in osteosarcoma (27). Notably, miR-138-5p or miR-422a may have a negative connection with hTERT in tumor cell proliferation and metastasis. Thus, combined with the results of our study, the probability of miR-138-5p and miR-422a potentially inhibiting hTERT expression in CRC is valuable. However, this results remains to be confirmed in future studies

Similar to previous studies showing the downregulation of miR-138-5p and miR422a in cancer tissues, in our study, we investigated whether or not the decreased levels of miR-138-5p and miR-422a in CRC were associated with the clinicopathology and survival of patients. A statistically significant difference in miR-138-5p expression was observed with regard to distant metastasis (P<0.000) while a significant difference in miR-422a expression was also noted between subgroups according to lymph-node metastasis (P=0.023). In addition, we found that the high- vs. low-expression group of miR-422a showed a highly significant difference in CRC patients (P=0.024), which suggests that the downregulation of miR-422a was associated with a poorer prognosis.

The present study had some limitations. First, immunohistochemistry was used to detect hTERT protein expression in CRC instead of western blotting. Second, in the present analysis, elevated levels of miR-422a expression were found to have a prognostic role in CRC, but it was not possible to confirm miR-422a as an independent predictive factor. Third, validation of miRNAs with the regulation of hTERT in CRC requires a cell function test, which is to be conducted in future studies.

In conclusion, our results confirm that miR-124-3p, miR-133a-3p, miR-133b, miR-138-5p, miR-150-5p, miR-378a-3p and miR-422a expression levels were downregulated in CRC and that miR-138-5p and miR-422a were found to potentially interact with hTERT. Investigation of the suppression of malignant behavior of these miRNAs in CRC may be useful as a diagnostic or prognostic tool at least, and may contribute to the development of a new effective treatment for CRC.

## Figures and Tables

**Table I:** The correlation between miR-138-5p and miR-422a expression and clinicopathological parameters in 84 colorectal cancer patients.

Parameters	Total (n=84)	miR-138-5p		P-value	miR-422a		P-value
		Low (n=43)	High (n=41)		Low (n=42)	High (n=42)	
Age (years)				0.166			0.450
<50	21	8	13		9	12	
≥50	63	35	28		33	30	
Gender				0.497			0.381
Male	46	22	24		21	25	
Female	38	21	17		21	17	
Tumor size (cm)				0.130			0.378
<5	36	15	21		20	16	
≥5	48	28	20		22	26	
Tumor site				0.257			0.659
Colon	48	22	26		23	25	
Rectum	36	21	15		19	17	
Differentiation				0.071			0.825
G1+G2	49	21	28		24	25	
G3+G4	35	22	13		18	17	
T				0.238			0.242
T1+T2	14	9	5		9	5	
T3+T4	70	34	36		33	37	
N				0.126			0.023^a^
Negative	30	12	18		10	20	
Positive	54	31	23		32	22	
M				0.000^a^			0.287
Negative	66	27	39		31	35	
Positive	18	16	2		11	7	
TNM stage				0.123			0.064
I+II	28	11	17		10	18	
III+IV	56	32	24		32	24	

Statistical analysis was performed by the χ^2^ test.

a P<0.05 was considered statistically significant.

**Table II:** PCR primer sequences for miRNAs and U6.

Genes	Forward primers (5′–3′)
hsa-miR-133a-3p	TTTGGTCCCCTTCAACCAGCTG
hsa-miR-133b	TTTGGTCCCCTTCAACCAGCTA
hsa-miR-422a	ACTGGACTTAGGGTCAGAAGG
hsa-miR-29c-3p	GCTAGCACCATTTGAAATCGGTTA
hsa-miR-124-3p	TAAGGCACGCGGTGAATG
hsa-miR-138-5p	GCAGCTGGTGTTGTGAATCA
hsa-miR-150-5p	CTCCCAACCCTTGTACCAGTG
hsa-miR-378a-3p	ACTGGACTTGGAGTCAGAAGGC
U6	CGCAAGGATGACACGCAAATTCGT

**Table III:** miRNAs levels of colorectal cancer and corresponding normal tissue groups using RT-qPCR.

microRNA	Median (25–75th)		P-value
	Cancer group	Normal group	
miR-29c-3p	0.193822 (0.096222–0.405425)	0.181904 (0.070936–0.449074)	0.0260
miR-124-3p	0.001082 (0.000298–0.003321)	0.008278 (0.002707–0.028471)	<0.0001
miR-133a-3p	0.019915 (0.004420–0.073774)	0.187506 (0.083645–0.456176)	<0.0001
miR-133b	0.001285 (0.000296–0.005439)	0.016779 (0.007922–0.043813)	<0.0001
miR-138-5p	0.002022 (0.00380–0.010544)	0.010097 (0.004016–0.025342)	<0.0001
miR-150-5p	0.018194 (0.007202–0.064146)	0.060581 (0.020490–0.197861)	<0.0001
miR-378a-3p	0.027426 (0.009510–0.114644)	0.318763 (0.145602–0.820874)	<0.0001
miR-422a	0.050255 (0.014860–0.134671)	0.273810 (0.117263–0.488233)	<0.0001

RT-qPCR, reverse-transcriptase quantitative polymerase chain reaction.

**Table IV:** The relationship between the expression of miRNAs and hTERT protein expression in colorectal cancer tissues.

Groups	Total (84)	hTERT				r	P-value
		− (24)	+ (34)	++ (16)	+++ (10)		
miR-124-3p						−0.147	0.183
Low	42	8	21	6	7		
High	42	16	13	10	3		
miR-133a-3p						−0.208	0.058
Low	42	9	17	8	8		
High	42	15	17	8	2		
miR-133b						−0.018	0.874
Low	42	11	19	6	6		
High	42	13	15	10	4		
miR-138-5p						−0.362	0.001^a^
Low	43	6	19	9	9		
High	41	18	15	7	1		
miR-150-5p						−0.185	0.092
Low	42	8	19	9	6		
High	42	16	15	7	4		
miR-378a-3p						−0.064	0.562
Low	42	9	21	7	5		
High	42	15	13	9	5		
miR-422a						−0.306	0.005^a^
Low	42	7	17	11	7		
High	42	17	17	5	3		

Statistical analysis was performed by the Spearman’s rank-order correlation coefficient.

a P<0.05 was considered to be statistically significant; hTERT, human telomerase reverse transcriptase.
